# Intracranial internal carotid aneurysm causing diplopia

**DOI:** 10.1186/1865-1380-4-56

**Published:** 2011-09-02

**Authors:** Brian T Kloss, Rahul Patel, Anne Marie Sullivan

**Affiliations:** 1 SUNY Upstate Medical University, Department of Emergency Medicine, NY, USA; 2 SUNY Upstate Medical University, NY, USA; 3 SUNY Upstate Medical University, Department of Radiology, NY, USA

## Abstract

Internal carotid intracranial aneurysms are a relatively rare form of intracranial aneurysm that presents with diplopia, retro-orbital pain and unilateral headaches. The symptoms are progressive and the diagnosis should be considered in a patient presenting with these complaints. Underlying hypertension and advanced age are specific risk factors.

## Case Report

**Figure 1:**
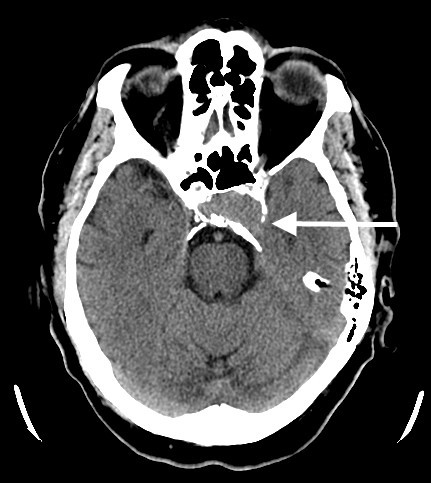
**CT without contrast demonstrates a soft tissue attenuation mass filling the sella with adjacent bone remodeling (arrow)**.

**Figure 2:**
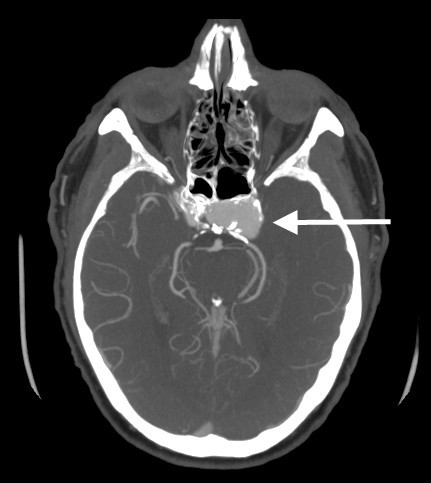
**On the post contrast images, there is a 1.8 × 2.9 × 1.6 cm (AP × transverse × craniocaudad) aneurysm with incomplete wall calcification originating from cavernous portion of the left internal carotid artery**.

**Figure 3:**
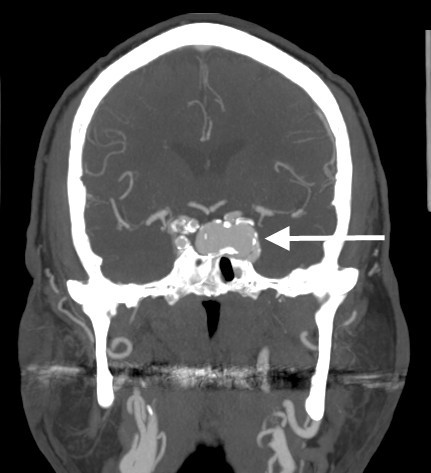
**On the post contrast images, there is a 1.8 × 2.9 × 1.6 cm (AP × transverse × craniocaudad) aneurysm with incomplete wall calcification originating from cavernous portion of the left internal carotid artery**.

**Figure 4:**
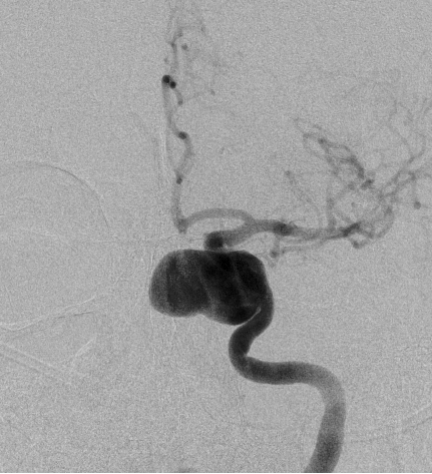
**Findings are confirmed by cerebral angiography and colorized**.

**Figure 5:**
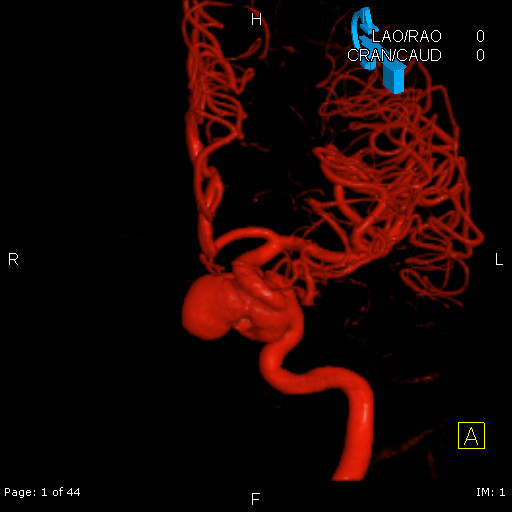
**Findings are confirmed by cerebral angiography and colorized**.

A 73-year-old diabetic male presented with progressively worsening diplopia and difficulty reading for 2 months. He denied having any fever, trauma, headache, numbness or weakness of his extremities or changes in other senses. Vital signs, physical and neurological exams were unremarkable, except for diplopia on right lateral gaze with distance, but not on left lateral gaze. CT, CTA, and MRI of the brain were obtained in addition to a cerebral angiogram (Figures 1, 2, 3, 4 and 5).

Internal carotid aneurysms located in the intracavernous region account for 3-5% of all intracranial aneurysms [1]. Systemic hypertension is a primary risk factor for development of aneurysm. Life-threatening risk or permanent neurological complications of most carotid cavernous aneurysms (CAA) are relatively low [2]. Despite this, ophthalmic morbidity is a leading consideration for treatment [3]. Diplopia (65% of cases), retro-orbital pain, and unilateral headache are the most common symptoms at presentation of CAA, followed by CN III and CN VI paresis [4]. The mainstay of symptomatic CAA has moved away from surgical and endovascular balloon techniques in favor of endovascular stenting and coiling approaches [4].

## Competing interests

The authors declare that they have no competing interests.

## Authors' contributions

BK oversaw the editorial aspects of the article and wrote the case presentation. RP wrote the section of disease presentation and management. AS reviewed and edited the clinical images. All authors read and approved the final manuscript.

## Consent

Patient consent was obtained and the case report qualified for IRB exemption given the lack of specific identifiable patient information within the case report and clinical images.
